# Achalasia and mental retardation in a child with uniparental disomy of *GMPPA* treated with POEM

**DOI:** 10.1093/gastro/goaf083

**Published:** 2025-09-19

**Authors:** Xiaoxia Qiu, Ziqing Ye, Ying Huang

**Affiliations:** Department of Gastroenterology, Children’s Hospital of Fudan University, Shanghai, P. R. China; Department of Gastroenterology, Children’s Hospital of Fudan University, Shanghai, P. R. China; Department of Gastroenterology, Children’s Hospital of Fudan University, Shanghai, P. R. China

## Introduction

Achalasia is a rare esophageal disorder with an incidence of 0.11 per 100,000 annually in children [1]. The most common syndrome involving achalasia is triple-A syndrome (OMIM#231550)—an autosomal recessive disorder characterized by adrenal insufficiency, achalasia, alacrima, and neurodegeneration [2]. Achalasia is identified in alacrima, achalasia, and mental retardation (AAMR) syndrome (OMIM#615510)—an autosomal recessive disease caused by loss-of-function variants in *GMPPA* [2]. There have been 22 patients reported [2–6]. AAMR and triple-A syndrome overlap in achalasia and alacrima. Absence of adrenal insufficiency in AAMR aids differentiation [5]. AAMR exhibits the involvement of multiple systems, such as muscular hypotonia and gait abnormalities [2]. Achalasia is treated with balloon dilatation, botulinum toxin injection, and Heller myotomy, and treatment has been revolutionized with per-oral endoscopic myotomy (POEM) [1]. POEM has become the mainstream treatment, with favorable outcomes compared with Heller myotomy and pneumatic dilation [1]. Inheritance of a homologous pair of chromosomes from one parent (segmental or total) results in uniparental disomy (UPD), which is associated with the imprinting and unmasking of autosomal recessive disorders [7].

Herein, we report an AAMR case with maternal UPD of chromosome 2 causing the homozygous mutation of *GMPPA*. There have been no previous reports of AAMR resulting from UPD. This is the youngest patient with AAMR receiving POEM.

## Case report

**Figure 1.**
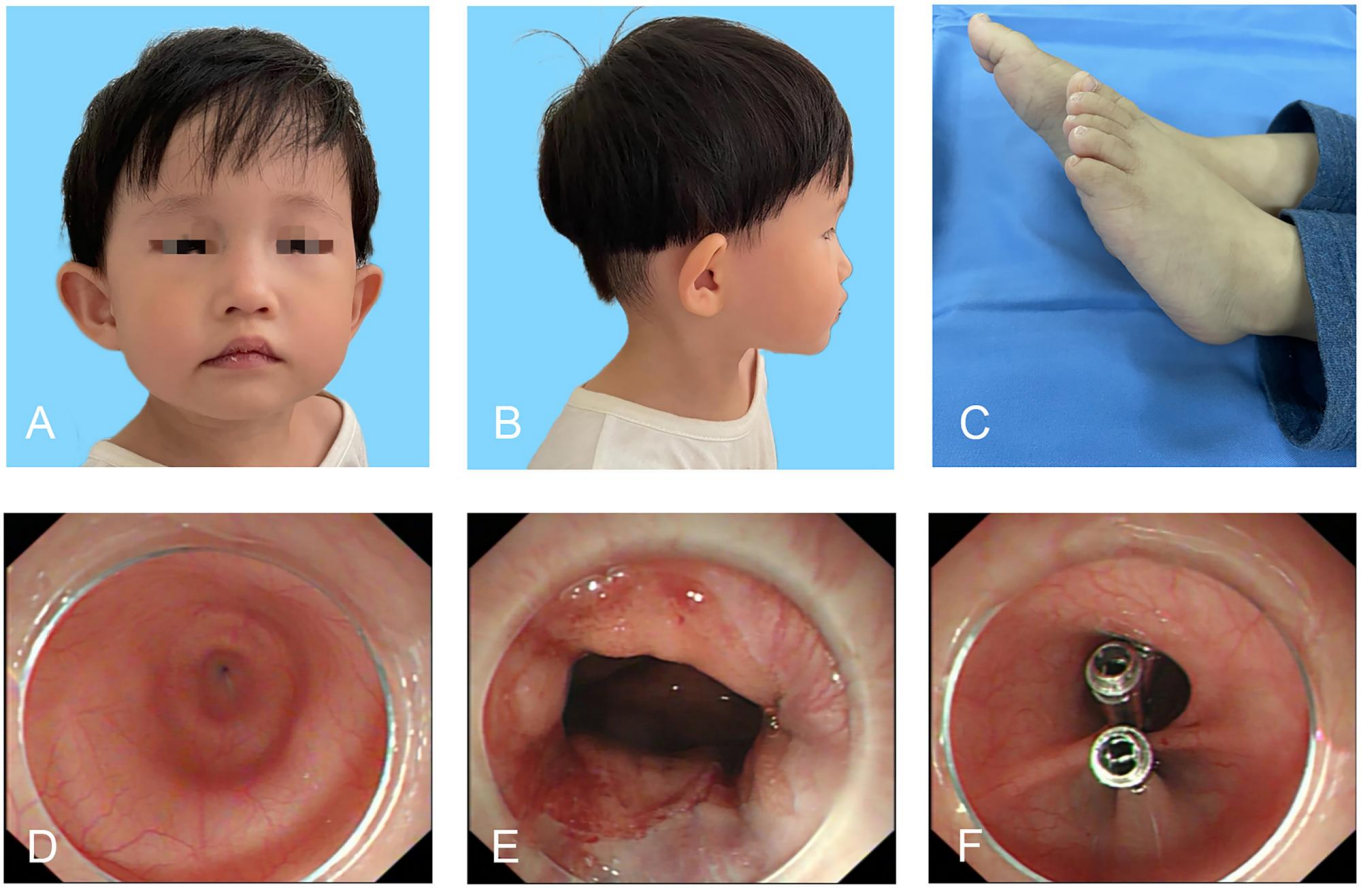
Clinical data of the patient. (A, B) Frontal and lateral views of the patient. (C) Lower-limb discrepancy. (D) Endoscopic image showing dilated esophagus before myotomy procedure. (E) Endoscopic image after myotomy procedure. (F) Endoscopic image showing closure of the mucosal entry with titanium clips.

The patient is a 2-year-old female. She is the second child, delivered vaginally at full term to nonconsanguineous Chinese parents. At birth, she presented with hypoxia. She had recurrent vomiting and gastroesophageal reflux since 6 months old. Despite formula changes and proton-pump inhibitors, her symptoms persisted. She had global developmental delay and pneumonia. She was diagnosed as having gastroesophageal reflux disease and underwent esophageal stricture at a local hospital. Investigations showed hypothyroidism, so levothyroxine was given. When presented to us, her height was 77 cm, weight 8 kg and BMI 13.5 kg/m^2^. She had dysphagia for solids, but could tolerate liquids. Upper endoscopy showed a dilated esophagus. Computed tomography revealed a dilated esophagus and pneumonia. Barium esophagram showed a “bird beak sign”, confirming achalasia. Evaluation confirmed alacrimia and dislocation of the left hip joint. Adrenal insufficiency was excluded. She was managed by a multidisciplinary team. The parents consented to POEM. Before POEM, the patient received parenteral nutrition and intravenous antibiotics for pneumonia. POEM was performed by using a gastroscope (GIF-Q260J; Olympus, Tokyo, Japan), a hybrid knife (ERBE; Erbe Elektromedizin, Tübingen, Germany), hot biopsy forceps (FD-410R; Olympus), and titanium clips (HX-610–90; Olympus, Tokyo, Japan). POEM was performed under general anesthesia and endotracheal intubation. An initial mucosal incision was performed on the posterior esophagus 5 cm above the esophagogastric junction (incisor-cardia distance: 23 cm). Myotomy was performed 4 cm above the esophageal-gastric junction and extended 1 cm into the cardia (Figure 1). During POEM, there was bloating in the right abdomen. As needle decompression did not show rushes of air, subcutaneous emphysema was diagnosed. Closure of mucosal entry was applied with eight titanium clips. The total operating time was 15 minutes. Post-operative X-ray excluded pneumothorax, pneumoperitoneum, and pneumomediastinum. A liquid diet was resumed on post-operative Day 3. She was discharged on post-operative Day 7. She was prescribed with a proton-pump inhibitor for 3 months. Restriction of dietary mannose was advised. She gained weight and was followed up. The Z score for weight improved from -7.0 (at admission) to -6.2 (1 month post-operative), -5.2 (7 months post-operative), and -4.6 (19 months post-operative). She did not experience catch-up growth due to unsuccessful hip surgery at another hospital. No recurrent vomiting and pneumonia were reported after POEM.

## Genetic sequencing

Trios whole exome sequencing showed that she was homozygous for *GMPPA* NM_205847.3 c.178C>T, p.(Gln60Ter). Sanger sequencing revealed that the mother was heterozygous for the mutation in *GMPPA*. Her father was wild-type. Trios whole exome sequencing data showed that variants on chromosome 2 were inherited from the mother, while loss of heterozygosity detected by B Allele Frequency confirmed the presence of maternal UPD of chromosome 2 [UPD(2)mat] [8]. The c.178C>T variant is not reported in the 1,000 Genome Project, Exome Aggregation Consortium or Genome Aggregation Database. The c.178C>T variant leads to a stop-codon mutation. She was diagnosed with AAMR caused by a *GMPPA* novel homozygous pathogenic variation with UPD(2)mat.

## Discussion and conclusions

The *GMPPA* gene encodes for guanosine diphosphate-mannose-pyrophosphorylase-A, which is an allosteric feedback inhibitor of GMPPB [9]. Loss-of-function variants in *GMPPA* cause hyperglycosylation and increased turnover of α-dystroglycan, leading to neuron degeneration and motor deficits [9].

A review of 23 cases showed that all had achalasia, alacrima, and delayed development. Patients with AAMR displayed heterogeneity with neurological symptoms (i.e. muscular hypotonia, gait abnormalities) and ocular symptoms (i.e. vision impairment, nystagmus) [2, 5]. Achalasia is the most debilitating symptom, causing gastroesophageal reflux, pneumonia, and failure to thrive. Most cases had feeding difficulty and pneumonia, requiring outpatient visits and hospitalizations [4, 6]. Among all cases with available data (*n *= 7), one patient underwent Heller myotomy at 8 months [5], two patients had surgery (not specified) at 2 months [6], one patient received laparoscopic Heller myotomy at 14 years [4], two patients had gastrostomy and POEM at 7 and 9 years, respectively [4], and one patient had fundoplication and gastrostomy at 10 weeks [6]. POEM was indicated when AAMR was diagnosed. She received POEM at age 2.9 years and was the youngest patient with AAMR to have received POEM. A review by the North American Societies of Pediatric Gastroenterology, Hepatology and Nutrition advocates that POEM is the mainstay of treatment for pediatric achalasia [1].

Patients with AAMR display multiple comorbidities [2]. Our patient had a dislocated hip joint. Indicated surgical reduction was postponed because of severe malnutrition. Skeletal abnormalities were reported in two siblings with AAMR, showing fusiform fingers, bilateral hypoplasia of thumbs, and brachydactyly of toes [10].

Fifteen mutations in *GMPPA* have been reported, including two frameshifts (p.Ala71Profs*19, p. Leu89fs), two nonsense mutations (p.Arg99Ter, p. Trp214Ter), nine missense mutations (p.Thr292Pro, p. Thr334Pro, p. Asn401Thr, p. Thr334Met, p. Arg390Pro, p. Gly182Asp, p. Arg373Pro, p. Gly308Arg, p. Arg318Trp), and two splicing mutations (c.993 + 1G>A, c.853 + 1G>A). There has been no report of UPD in *GMPPA*.

In summary, we described the first AAMR case of UPD(2)mat with a novel homozygous variant in *GMPPA*. Multidisciplinary management is required. Prompt POEM leads to promising outcomes.

## Authors’ contributions

X.Q. conceptualized and designed the study, drafted the initial manuscript, and critically reviewed and revised the manuscript. Z.Y. and Y.H. designed the data-collection instruments, collected data, carried out the initial analyses, and critically reviewed and revised the manuscript. All authors read and approved the final version of the manuscript.
